# Multi-Modal Metabolomics Deciphers Pan-Cancer Metabolic Landscapes and Spatial-Niche-Specific Alternations

**DOI:** 10.3390/metabo16020129

**Published:** 2026-02-13

**Authors:** Tingze Feng, Hai-Long Piao, Di Chen

**Affiliations:** 1 State Key Laboratory of Phytochemistry and Natural Medicines, Dalian Institute of Chemical Physics, Chinese Academy of Sciences, 568 Lvshunzhong Road, Dalian 116051, China; tingzef@dicp.ac.cn; 2 University of Chinese Academy of Sciences, Beijing 100049, China; 3 Department of Thoracic Surgery, Cancer Hospital of Dalian University of Technology, Liaoning Cancer Hospital & Institute, Shenyang 110042, China

**Keywords:** spatial metabolomics, tumor microenvironment, pan-cancer, carnitine

## Abstract

Background: Metabolic reprogramming is a hallmark of cancer and supports tumor growth and adaptation within the tumor microenvironment (TME). The complexity of this reprogramming manifests as both distinct variations across cancer types and spatial heterogeneity within individual tumors. The specificity of these metabolic alterations, whether to cancer type, spatial niche, or as shared features, remains unclear, highlighting a critical gap in our systematic, pan-cancer understanding of metabolic reprogramming. Methods: We integrated bulk metabolomics and spatial metabolomics to investigate pan-cancer metabolic features and used blood-based metabolomics and spatial transcriptomics data to validate key findings. Metabolic differences were compared between tumor and normal tissues across multiple cancer types at the bulk level to identify metabolic modules shared across cancers or specific to individual cancer types. A two-step clustering framework was applied to identify both local and global TME-associated spatial metabolic modules of spatial metabolomics data from various tumor tissue slices. Results: We have identified a spectrum of metabolic features, including those specific to individual cancer types or spatial architectures and others shared across cancers, with some features emerging only at bulk-level and others uniquely discernible through spatial metabolomics. Integrative analyses also identified 19 metabolites consistently altered in both bulk and spatial data, especially carnitine species, which also showed concordant changes in blood samples and spatial associations with genes involved in fatty acid metabolism. Conclusions: This pan-cancer, multi-scale integrative analysis highlights substantial metabolic heterogeneity within the TME and across cancer types and identifies metabolites with consistent alterations across analytical layers, providing candidate features for future studies of tumor metabolism and potential metabolic biomarkers.

## 1. Introduction

The tumor microenvironment (TME) is a complex and dynamic ecosystem composed of malignant cells, surrounding immune cells, stromal cells, and vascular components that continuously interact [1,2,3]. Metabolic reprogramming has been recognized as a fundamental process supporting this complex ecosystem [4]. Tumor cells can reprogram multiple metabolic pathways, such as in the Warburg effect, glycolysis, fatty acid synthesis and oxidation, and the tricarboxylic acid cycle, to adapt to unfavorable conditions, including proliferation, nutrient limitation, and hypoxia [3,5,6]. Moreover, numerous prior studies have reported aberrant metabolic processes in specific tumor types [7,8,9,10,11,12]. Although metabolic dysregulation is widely regarded as a hallmark of cancer [13,14], it has not been systematically compared at the pan-cancer level, and its spatial organization within the tumor microenvironment remains insufficiently characterized.

With advances in spatial omics technologies, spatial metabolomics has emerged as a powerful approach to investigating metabolic heterogeneity in tumors [15,16]. Unlike conventional bulk metabolomics, which captures only tissue-averaged metabolic states, spatial metabolomics enables the direct measurement of metabolite distributions within tissue sections while preserving spatial context, thereby revealing metabolic differences across distinct spatial regions [17]. This capability allows for characterization of metabolic features in tumor boundaries, tumor cores, and other specific TME niches [18,19]. Previous studies applying spatial metabolomics to individual cancer types reported heterogeneous distributions of lipid species and energy metabolism-related molecules across tumor regions, suggesting that spatial metabolic features may be associated with tumor invasion, growth, and adaptation to the TME [20,21,22,23]. However, current applications of spatial metabolomics primarily focus on single cancer types or limited sample sizes and describe localized observations, leaving a lack of systematic, pan-cancer comparisons of metabolic patterns across cancer-type-specific or spatial-niche-specific contexts.

This study took metabolites themselves as the analytical starting point and integrated bulk metabolomics with spatial metabolomics, further incorporating blood-based metabolomics and spatial transcriptomics data, to systematically characterize tumor metabolic reprogramming at both tissue-averaged and spatially resolved levels from a pan-cancer perspective. Specifically, we first compared metabolic differences between tumor and normal tissues across multiple cancer types at the bulk level to identify metabolic modules that were shared across cancers or specific to individual cancer types. Subsequently, to characterize differential spatial distributions of distinct members within the same category of metabolites across tumor regions, we analyzed spatial metabolite profiles from multiple tumor tissue slices to identify TME-associated spatial metabolic modules (SMMs). Finally, we focused on metabolites that exhibited aberrant changes in both bulk and spatial metabolomics analyses and integrated blood-based metabolomics and spatial transcriptomics data to evaluate the consistency of these metabolic alterations. Through this pan-cancer, multi-scale analytical framework, this study aimed to provide a systematic perspective on metabolic heterogeneity within the TME and across cancer types. In addition, it sought to identify candidate leads for future investigations into metabolic mechanisms and potential biomarkers.

## 2. Materials and Methods

### 2.1. Collection of Pan-Cancer Bulk and Spatial Metabolomics Datasets

We assembled four tissue-based bulk metabolomics datasets for kidney [24], breast [24,25], ovarian [26], and brain cancers [24,27]. In total, these datasets included 314 samples, comprising 208 tumor tissues and 106 matched or adjacent normal tissues. In addition, we collected spatial metabolomics datasets for six cancer types, including kidney, breast, ovarian [28], brain, stomach cancer [29] and head and neck squamous cell carcinoma (HNSCC), comprising 19 tissue slices in total: seven stomach cancer slices, five kidney cancer slices, four HNSCC slices, three ovarian cancer slices, and two slices each for breast and brain cancers. For each cancer type, the spatial metabolomics data were derived from at least two independent patient specimens. All spatial metabolomics datasets were downloaded from the METASPACE database platform (https://metaspace2020.org/ (accessed on 9 October 2025)) [30]. More detailed information on all bulk and spatial metabolomics datasets is provided in Supplementary Table S1. Blood-based metabolomics data were downloaded from the MACdb database (https://ngdc.cncb.ac.cn/macdb/ (accessed on 20 December 2025)) [31,32]; the detailed results are provided in Supplementary Table S2.

### 2.2. Collection of Metabolite Annotation

We downloaded the XML file containing all currently available metabolite annotations from the Human Metabolome Database (HMDB, version 5.0) [33]. For each HMDB entry, we parsed the following fields: accession, name, secondary_accessions, formula, kegg_id, wikipedia_id, metlin_id, pubchem_compound_id, chebi_id, direct_parent, super_class, class, and sub_class. Among these, super_class, class, sub_class, and direct_parent represented the hierarchical metabolite classification terms. Super_class corresponded to the highest-level category, whereas class, sub_class, and direct_parent provided increasingly specific sub-classifications for a given metabolite. To harmonize metabolite identifiers across datasets, we generated an additional column termed “merged_accession”, in which the primary HMDB accession was combined with all associated secondary_accessions. This step ensured that metabolites could be consistently annotated even when different datasets used either the primary HMDB accession or one of the secondary accessions as the metabolite identifier. The curated metabolite annotation table is summarized in Supplementary Table S3.

### 2.3. Bulk Metabolomics Data Analysis

We first performed differential metabolite analyses on the collected bulk metabolomics datasets to compare metabolite abundance between tumor and normal tissues across four cancer types: breast cancer, kidney cancer, brain cancer, and ovarian cancer. For the ovarian cancer dataset, metabolite abundances were log-transformed before differential analysis. The bulk metabolomics data for the other three cancer types had already undergone preprocessing in the original studies and were therefore directly used for differential analyses [24]. For each cancer type, differences in metabolite abundances between tumor and normal samples were assessed using the Wilcoxon rank-sum test (Wilcox test) to determine whether the distributions of metabolite abundances differed significantly between the two groups. *p* values were subsequently adjusted, and metabolites with an adjusted *p* value < 0.05 and an absolute log fold change (logFC) greater than zero were defined as significantly differentially abundant metabolites. Based on these criteria, a total of 121, 336, 229, and 46 significantly differentially abundant metabolites were identified in breast cancer, kidney cancer, brain cancer, and ovarian cancer samples, respectively (Supplementary Table S4). Given the substantial differences in metabolite coverage across cancer cohorts, we further focused on metabolites that met at least one of the following criteria to systematically identify cross-cancer bulk metabolic modules: metabolites detected in all four cancer types or metabolites identified as significantly differentially abundant in at least three cancer types. Based on this filtering strategy, 38 metabolites were retained for subsequent cross-cancer analyses. These metabolites were further classified according to the direction of their abundance changes across the four cancer types (upregulated, downregulated, not significantly changed, or not detected), and four bulk metabolic modules (BMMs) were defined accordingly.

### 2.4. Spatial Metabolomics Data Analysis

We first performed quality control and preprocessing on the collected spatial metabolomics data. Specifically, spatial spots with zero values across all metabolites were removed, and metabolites with zero values in more than 80% of spatial spots were excluded to reduce data sparsity and its impact on downstream analyses. In addition, spatial spots corresponding to apparent background or experimental contamination signals were manually identified and removed. After data filtering, missing values of the remaining metabolites were imputed. Missing value imputation was performed using the GCN Imputation method developed in our previous study to improve data completeness and reduce bias introduced by random missingness. Subsequently, for each tissue slice, spatial clustering was performed independently within each slice based on the imputed spatial metabolite abundance matrix, using the general unsupervised analysis workflow implemented in the R package Seurat (version 5.1.0) [34]. This clustering analysis was conducted separately for each sample to identify local spatial clusters within the same tissue slice that exhibited similar metabolic compositions. To compare metabolite abundances between each spatial cluster and the remaining clusters, metabolites showing significantly higher abundance in a given spatial cluster in at least two different cancers were identified through differential analyses within each tissue slice. Notably, the first-step clustering and differential analyses were performed independently for each slice, and cluster identities were not assumed to be directly comparable across different samples. Based on these results, we performed a second-step clustering of all the local spatial clusters to capture distinct SMMs among all tissue slices at a global level, analyzing similarity patterns of metabolite markers of all the local spatial clusters across samples.

### 2.5. Statistical Analysis

All statistical analyses and data visualization were performed in the R software environment (version 4.2.0) and Python (version 3.10.16). Data visualization was primarily conducted using the R packages ggplot2 (version 3.4.2) [35], ggpubr (version 0.4.0), pheatmap (version 1.0.12), patchwork (version 1.3.2) [36], and ggstatsplot (version 0.9.5) [37].

## 3. Results

### 3.1. Identification of Shared and Cancer-Specific Metabolic Modules Across Cancers Using Bulk Metabolomics Datasets

**Figure 1:**
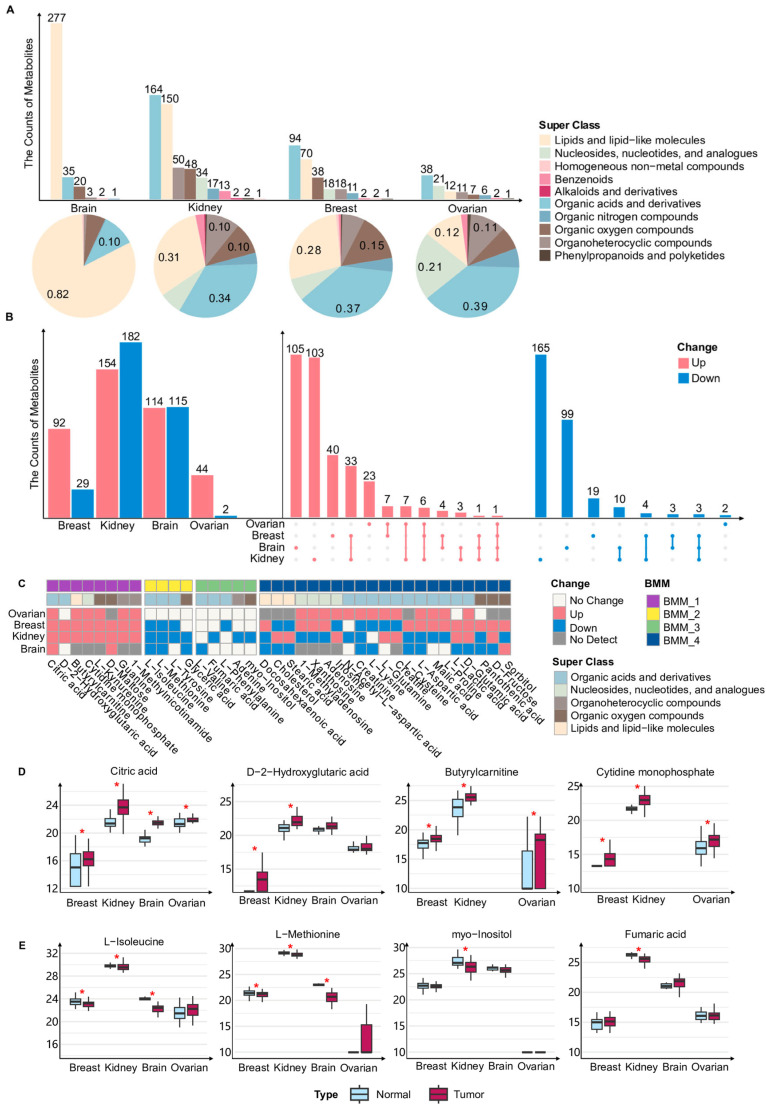
Overview of bulk metabolomic alterations and metabolic modules across multiple cancers. (**A**) Bar and pie charts depict the distribution of metabolite super classes in bulk metabolomics of ovarian, breast, kidney, and brain cancers. The upper bar plot shows the number of metabolites belonging to each class in each cancer type. The lower pie charts illustrate the relative proportion of each metabolite class within the four cancers. Metabolite classification was based on the super class annotation provided by the Human Metabolome Database (HMDB). (**B**) The bar plots on the left show the numbers of metabolites that were significantly upregulated (red) or downregulated (blue) in tumor versus normal tissue for each cancer type. The upset plot on the right summarizes the intersections of these differential metabolites across the four cancers and displayed metabolites that were uniquely dysregulated in a single cancer, as well as those that were consistently upregulated or downregulated across four cancers. (**C**) The heatmap of four bulk metabolic modules identified from the bulk metabolomics of four cancers. BMM: Bulk Metabolic Module. (**D**) Box plots show the abundance of metabolites of BMM_1 in tumor and normal samples from four cancers. Red asterisks indicate metabolites with adjusted *p* values < 0.05 based on the Wilcoxon rank-sum test. (**E**) Box plots show the abundance of metabolites of BMM_2 (left two box plots) and BMM_3 (right two box plots) in tumor and normal samples from four cancers. Red asterisks indicate metabolites with adjusted *p* values < 0.05 based on the Wilcoxon rank-sum test.

We first summarized and classified all metabolites in the bulk metabolomics datasets from ovarian, breast, kidney and brain cancers, using metabolite annotations based on the super class defined in the HMDB database. These metabolites were assigned to 10 super class categories, with the majority belonging to lipid and lipid-like molecules, organic acids and derivatives, organic oxygen compounds, and nucleosides, nucleotides, and analogues (Figure 1A). In the brain cancer dataset, approximately 82% of detected metabolites were classified as lipid and lipid-like molecules, representing a markedly higher proportion than in the other three cancer types (Figure 1A). In contrast, metabolites detected in ovarian, breast, and renal cancers were predominantly enriched in the organic acids and derivatives category (Figure 1A). We next compared metabolites exhibiting statistically significant abundance differences between tumor and normal tissue samples within each of the four cancer types (Figure 1B). Most differentially abundant metabolites displayed pronounced cancer-type specificity and were identified in only a single cancer type (Figure 1B). Meanwhile, several metabolites showed concordant changes across multiple cancers (Figure 1B). It should be noted that variations in the composition of detected metabolites across cancer types may arise from both biological factors, such as tissue origin, and technical factors, including differences in sample processing workflows and metabolomics platforms. Consequently, these differences may reflect a combination of biological heterogeneity and technical variability across datasets.

To systematically characterize these cross-cancer metabolic patterns and ensure comparability across platforms, we focused on metabolites that were either detected in all four cancers or identified as differentially abundant in at least three cancer types. Based on the direction of their abundance changes in tumor tissues relative to normal tissues, these metabolites were grouped into four BMMs (Figure 1C). BMM_1 comprises metabolites that were generally more abundant in tumor tissues. Notably, citric acid exhibited an increased abundance across all four cancer types, consistent with prior evidence that intracellular citrate is essential for tumor cell survival and expansion (Figure 1C,D) [38]. Butyrylcarnitine showed a higher abundance in tumor tissues compared with normal tissues across multiple cancers (Figure 1C,D) [39,40]. BMM_2 includes metabolites that were generally less abundant in tumors, among which L-methionine was consistently reduced in breast, renal, and brain cancers (Figure 1C,E). In line with previous observations, elevated methionine cycle activity in tumor-initiating cells can lead to methionine consumption exceeding its regeneration [41]. BMM_3 contains metabolites that were detected in all four cancers but showed significant changes only in a single cancer type, reflecting highly cancer-specific metabolic adaptations (Figure 1C). BMM_4 comprises metabolites that were differentially abundant in multiple cancers but exhibited inconsistent directions of change across cancer types (Figure 1C), suggesting that these metabolites may play self-sufficient roles in distinct TMEs. Overall, bulk metabolomics analyses reveal a dual pattern of metabolic reprogramming across cancers. Among them, several metabolites showed consistent alterations across multiple cancer types, potentially reflecting conserved metabolic demands during tumor progression, but a large proportion of metabolites displayed pronounced cancer-type-specific changes, highlighting the substantial diversity of metabolic regulation across different cancers.

### 3.2. Identification of Spatial Metabolic Modules Across Heterogeneous Tumor Tissues

**Figure 2:**
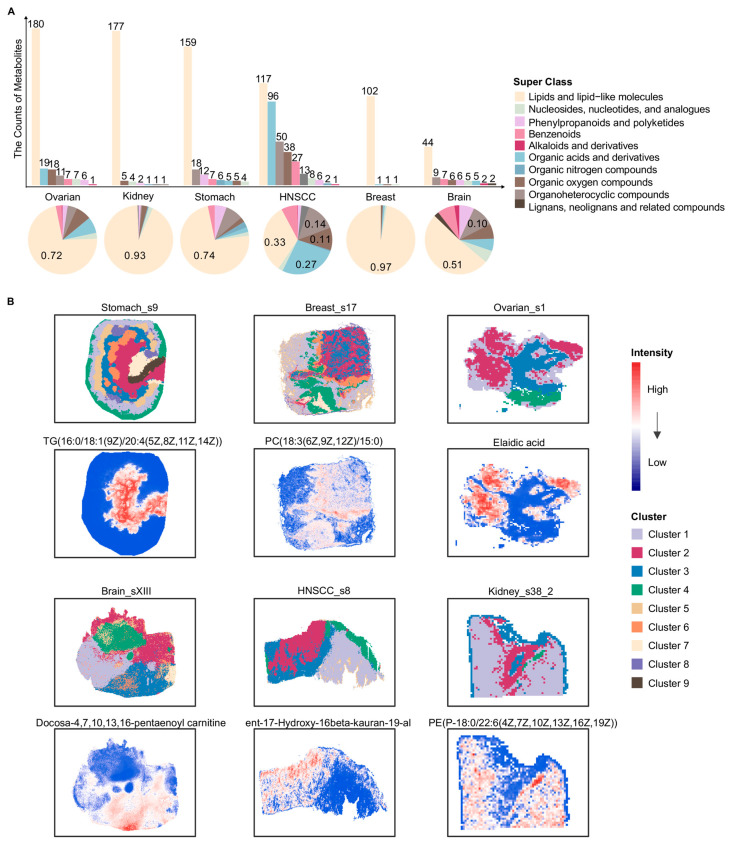
Overview of spatial metabolite classes and local spatial clusters across six cancers. (**A**) Bar and pie charts depict the distribution of metabolite super classes detected by spatial metabolomics in six different cancer types. The upper bar plot shows the number of metabolites assigned to each super class in each cancer type. The lower pie charts illustrate the relative proportion of each metabolite super class within each cancer. Metabolite classification was based on the super class annotation provided by HMDB. (**B**) For each of the six cancers, one representative tissue slice is shown with its local spatial clustering result and the spatial intensity distribution of one metabolite. For each slice, local clusters were defined independently based on their own spatial metabolomic profiles, and the cluster identities and colors were therefore not directly comparable across different cancer types or samples. HNSCC: Head and neck squamous cell carcinoma; TG: Triglyceride; PC: Phosphatidylcholine; PE: Phosphatidylethanolamine. Lipid species are annotated as fatty acyl composition (carbon chain length: number of double bonds).

Consistent with the bulk metabolomics data, we first summarized and classified the metabolites in spatial metabolomics tissue slices from six different cancer types: stomach, breast, ovarian, brain, HNSCC and kidney (Figure 2A). The number of metabolites was smaller compared with bulk metabolomics, while the associated super class still covered ten categories (Figure 2A). The metabolites were predominantly classified as lipid and lipid-like molecules across 19 slices (Figure 2A). To characterize the spatial distribution patterns of metabolites within tumor tissues, all slices underwent data filtering, missing-value imputation, and unsupervised clustering analysis. As shown in Figure 2B, clustering based on metabolite abundances effectively distinguished distinct spatial metabolite clusters. Each local spatial cluster identified within a tumor sample delineated a unique spatial metabolic pattern driven by a specific repertoire of metabolites (Figure 2B). These patterns reflect distinct, localized adaptations of tumor metabolism that likely underpin specific functional states or TME niches within the tissue architecture. In the slice Ovarian_s1, elaidic acid exhibited a relatively higher abundance in Cluster 2 (Figure 2B). Previous studies have reported that elaidic acid can promote the proliferation and migration of epithelial ovarian cancer (EOC) cells, and its abundance has been positively associated with the risk of EOC [42,43]. In the Breast_s17 slice, PC(18:3(6Z,9Z,12Z)/15:0) showed the highest abundance in Cluster 6 (Figure 2B). Phosphatidylcholines (PCs) have been linked to oncogene-activated signaling pathways and cellular differentiation processes involved in tumor progression and metastasis [44,45]. In the slice Stomach_s9, TG(16:0/18:1(9Z)/20:4(5Z,8Z,11Z,14Z)) exhibited the highest abundance in Cluster 2 (Figure 2B). Prior studies reported that high triglyceride (TG) levels were significantly correlated with poor outcomes in stomach cancer patients [46]. In the slice Kidney_s38_2, PE(P-18:0/22:6(4Z,7Z,10Z,13Z,16Z,19Z)) showed the highest abundance in Cluster 4 (Figure 2B). Phosphatidylethanolamines (PEs) have been linked to dysregulated metabolic processes in kidney tumors [28]. In addition, docosa-4,7,10,13,16-pentaenoyl carnitine and ent-17-Hydroxy-16β-kauran-19-al displayed pronounced spatial abundance patterns in the Brain_sXIII and HNSCC_s8 slices, respectively, further reflecting spatial metabolic heterogeneity across tumor types.

**Figure 3:**
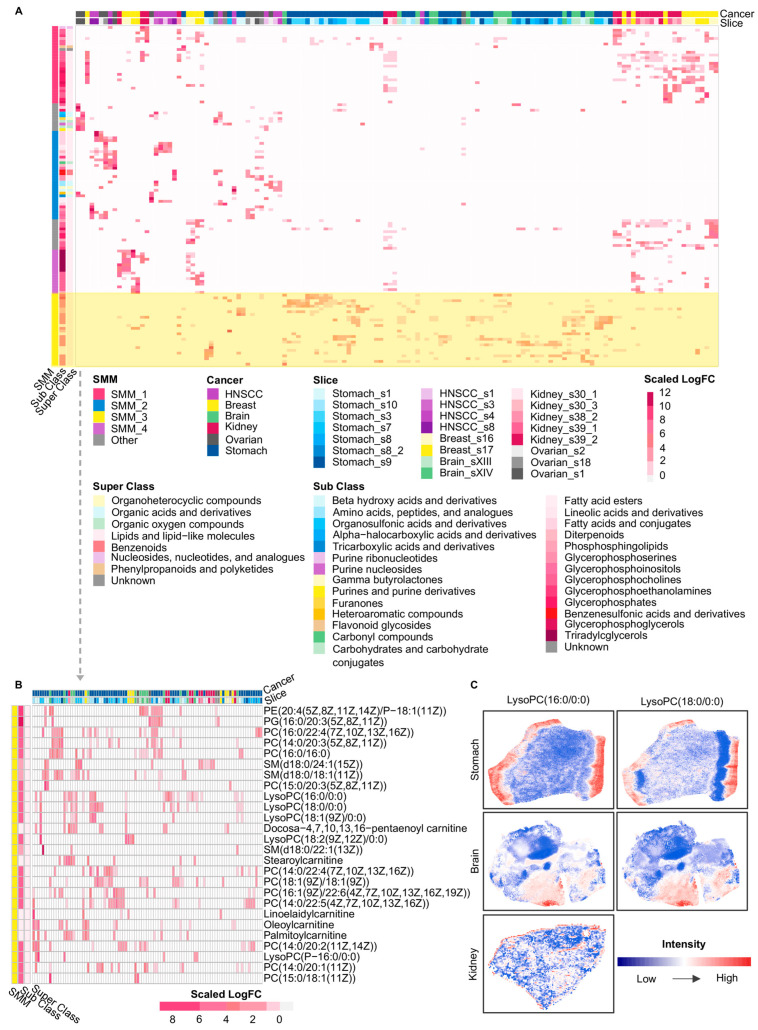
Overview of cross-cancer spatial metabolic modules and representative spatial patterns. (**A**) Heatmap of SMMs across slices and cancer types. Columns: spatial clusters; rows: metabolites. The color of the heatmap represents the scaled logFC values of metabolites for each spatial cluster. Metabolites were annotated by HMDB super class and sub class, with color schemes indicating cancer type, tissue slice, and HMDB classifications. (**B**) The enlarged part of SMM_3 in the heatmap of A. Spatial clusters in this heatmap contain at least one metabolite of SMM_3 with the logFC greater than 0. (**C**) The spatial intensity of representative metabolites from SMM_3 in three cancers. HNSCC: Head and neck squamous cell carcinoma; SMM: Spatial Metabolic Module; PE: Phosphatidylethanolamine; PG: phosphatidylglycerols; PC: Phosphatidylcholine; SM: Sphingomyelin; LysoPC: Lysophosphatidylcholine. Glycerophospholipids are annotated based on fatty acyl composition (carbon chain length:number of double bonds), whereas sphingomyelins are annotated by sphingoid base and N-acyl chain composition.

Using the marker metabolites’ logFC, we performed a second hierarchical clustering to integrate all local spatial clusters and to identify spatial-niche-specific patterns, which revealed four SMMs across cancer types (Figure 3A). Representative metabolites within these spatial modules were predominantly lipid and lipid-like molecules, with a proportion belonging to organic acids and derivatives, organic oxygen compounds, and related categories (Figure 3A). Specifically, SMM_1 mainly consisted of phospholipid metabolites jointly enriched in HNSCC, breast cancer, brain cancer, kidney cancer, and stomach cancer, including phosphatidic acids (PAs), PCs, phosphatidylglycerols (PGs), and PEs (Supplementary Figure S1). SMM_2 was the only spatial module that concurrently included organic acids, organic oxygen compounds, and organoheterocyclic compounds, and this module was primarily observed in the ovarian cancer and HNSCC samples (Supplementary Figure S2). SMM_3 comprised PCs, lysophosphatidylcholines (lysoPCs), and carnitine-related metabolites enriched across breast, brain, kidney, stomach, and ovarian cancers (Figure 3B). Further spatial analysis showed that lysoPCs were mainly distributed at tissue boundary regions (Figure 3C), suggesting a potential association between their spatial localization and metabolic activities at tumor margins. SMM_4 was mostly composed of metabolites enriched in breast and kidney cancers, among which TGs exhibited prominent aggregation exclusively in these two cancer types (Supplementary Figure S3). Notably, SMM_1 and SMM_3 were both dominated by phospholipid-related metabolites, particularly PCs, yet they occupied distinct spatial regions within tumor tissues (Supplementary Figure S4). This observation indicates that even metabolites belonging to the same sub-class could pertain to different spatial-niche-specific patterns depending on metabolic demands within distinct tumor regions. For example, distinct members within the same phospholipid family, PC(15:0/16:0) in SMM_1 and PC(18:1(9Z)/18:1(9Z)) in SMM_3, exhibited distinct spatial abundance patterns within slice Stomach_s8 (Supplementary Figure S4), which suggests that high-resolution metabolite detection and detailed molecular annotation were essential for accurately resolving metabolites that function in different spatial regions of tumors. However, resolution levels in most transcriptomics-based or other omics studies of tumor metabolic reprogramming remained limited.

### 3.3. Identification of Shared Spatial Marker Metabolites Across Different Cancers

**Figure 4:**
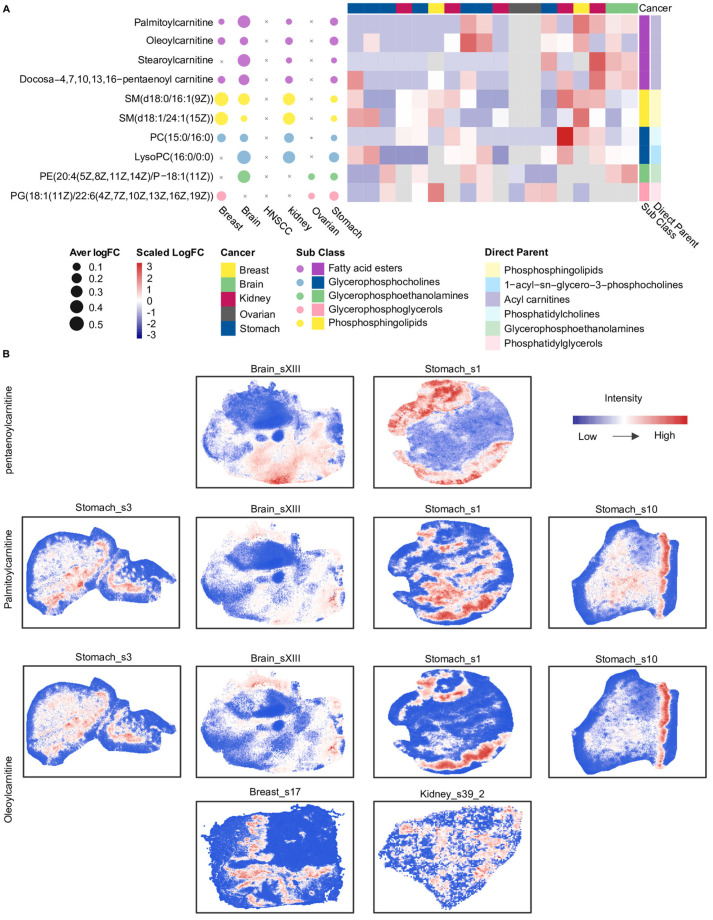
Overview of shared spatial metabolite markers and acyl−carnitine distributions across cancers. (**A**) The bubble plot on the left displays these 10 marker metabolites, where bubble size represents the mean logFC of each metabolite for each cancer type. The heatmap on the right shows the mean logFC of the 10 metabolites across all slices from five cancers; slices from HNSCC are not included because these metabolites were not detected in HNSCC. The heatmap was annotated using two levels of metabolite classification, including sub class and direct parent. The colors used for bubbles in the left panel match those used for the subclass annotation in the right panel. (**B**) Spatial intensity maps illustrate the distributions of three carnitine-related metabolites: docosa−4,7,10,13,16-pentaenoyl carnitine, palmitoylcarnitine, and oleoylcarnitine. Pentaenoylcarnitine: docosa−4,7,10,13,16−pentaenoyl carnitine. HNSCC: Head and neck squamous cell carcinoma; SM: Sphingomyelin; PC: Phosphatidylcholine; LysoPC: Lysophosphatidylcholine; PE: Phosphatidylethanolamine; PG: phosphatidylglycerols. Glycerophospholipids are annotated based on fatty acyl composition (carbon chain length:number of double bonds), whereas sphingomyelins are annotated by sphingoid base and N−acyl chain composition.

Next, we further identified 10 metabolites that were consistently classified as relatively highly abundant within specific spatial clusters across more than three cancer types (Figure 4A). Notably, four of these metabolites belonged to SMM_3 and were carnitine-related, including palmitoylcarnitine, oleoylcarnitine, stearoylcarnitine, and docosa-4,7,10,13,16-pentaenoyl carnitine, indicating a recurrent spatial enrichment of carnitine-related metabolites across multiple tumor types (Figure 4A). Further spatial distribution analysis showed that palmitoylcarnitine, oleoylcarnitine, and stearoylcarnitine exhibited similar spatial distribution patterns across different cancer types (Figure 4B). For instance, in the Stomach_s10 slice, palmitoylcarnitine and oleoylcarnitine displayed highly concordant intensity distributions. In contrast, docosa-4,7,10,13,16-pentaenoyl carnitine exhibited spatial distribution patterns distinct from palmitoylcarnitine and oleoylcarnitine in the Stomach_s1 and Brain_sXIII slices. The spatial intensity distributions among these carnitine metabolites may be related to differences in their associated metabolic processes.

### 3.4. Integrative Analysis of Bulk and Spatial Metabolomics Identifies Metabolites with Concordant Alteration

**Figure 5:**
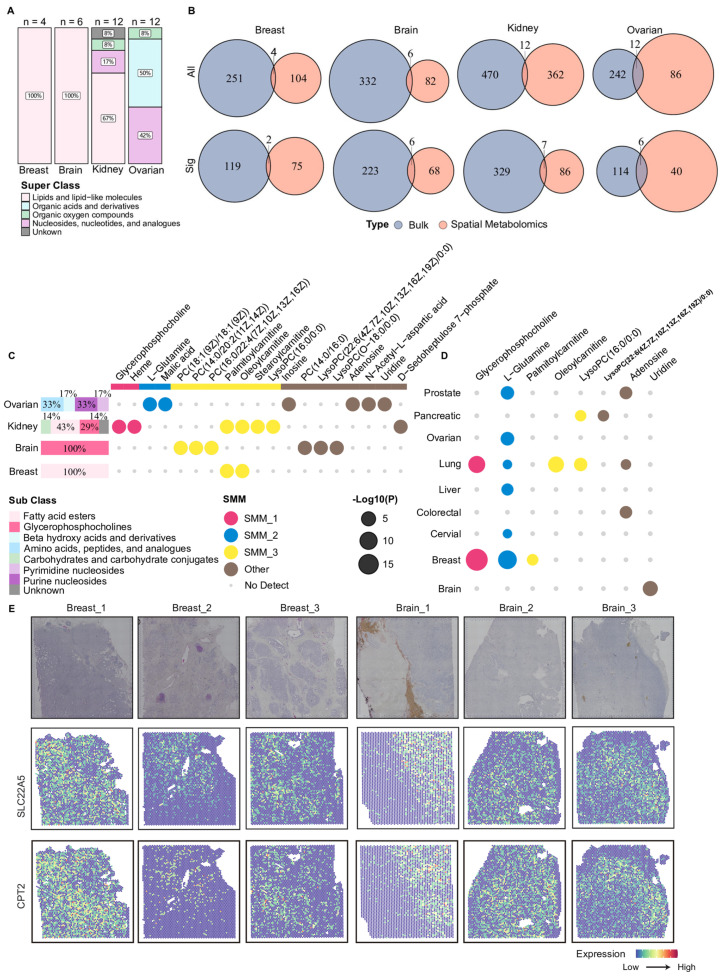
Overview of shared metabolites between bulk and spatial metabolomics. (**A**) The stacked bar plot of metabolites detected in both bulk and spatial metabolomics data. The proportional distribution displays HMDB metabolite super classes among these overlapping metabolites in each cancer type. (**B**) Venn plots compare metabolites measured in bulk (blue) and spatial (orange) metabolomics in each cancer type. The top row (“All”) shows the number of all metabolites detected. The bottom row (“Sig”) shows the number of significant metabolites. (**C**) The horizontal stacked bar plots on the left summarize the proportions of HMDB sub classes among significantly altered metabolites in both bulk and spatial metabolomics. The dot plot on the right indicates in which cancers the metabolite displayed remarkable changes in both. The color of the dot denotes the SMM or others to which it belonged and whether the metabolite had been detected or not. (**D**) Bubble plot showing the statistical significance of eight metabolites in blood-based metabolomics data across nine cancer types. Bubble size represents the level of statistical significance. Bubble colors denote the corresponding SMMs to which each metabolite was assigned. (**E**) H&E staining images and spatial expressions of two carnitine-related enzymes, SLC22A5 and CPT2, for three breast cancer and three brain cancer tissues. Color intensity indicates normalized gene expression levels across spatial spots. SMM: Spatial Metabolic Module.

Building on the above analyses, we systematically extracted the overlap between metabolites detected by bulk and spatial metabolomics, as well as their corresponding differentially abundant metabolites, to identify metabolites that exhibited concordant changes at both the tissue-averaged and spatially resolved levels (Figure 5A,B, Supplementary Table S4). We observed that only 34 metabolites intersected bulk and spatial metabolomics (Figure 5B top row). Given these platform-specific constraints, it is noteworthy that our integrated analysis nonetheless identified 19 metabolites showing concordant differential abundance in both bulk and spatial data, highlighting a robust overlap (Figure 5B bottom row and C). Notably, 11 of these 19 metabolites had previously been assigned to spatial metabolic modules, indicating that they not only exhibited changes at the overall tissue level but also displayed distinct spatial distribution patterns (Figure 5C). Several carnitine-related metabolites, including palmitoylcarnitine, oleoylcarnitine, and stearoylcarnitine, were consistently identified as differentially abundant in both bulk and spatial metabolomics analyses (Figure 5C). In particular, palmitoylcarnitine and oleoylcarnitine exhibited significantly higher abundance in tumor tissues from kidney cancer and breast cancer in the bulk metabolomics data (Figure 5C). This repeated observation across cancer types and analytical platforms further supported the presence of consistent alterations in these carnitine metabolites across multiple tumors.

To further support consistency across metabolomics types, we examined the changes in these 19 metabolites in blood-based metabolomics data (plasma or serum) through the MACdb database. Eight metabolites were also reported to be more abundant in blood samples from corresponding cancer patients (Figure 5D). Palmitoylcarnitine and oleoylcarnitine showed significantly higher abundances in serum samples from patients with breast cancer and lung cancer, respectively (Figure 5D). This finding indicated that the observed metabolic alterations were not restricted to tumor tissues but also detectable in circulation, suggesting concordant changes across tissue compartments. To further explore potential cellular processes associated with the altered carnitine metabolites at the molecular level, we integrated spatial transcriptomics data and examined the spatial expression patterns of key genes involved in carnitine metabolism. As shown in Figure 5E, relatively higher expression levels of SLC22A5 and CPT2 were observed in specific tumor regions in three breast cancer samples and one brain cancer sample. Overall, this study identified a set of metabolites that exhibited concordant alterations across multiple analytical layers, particularly carnitine-related metabolites, by integrating tissue-level bulk and spatial metabolomics, circulating blood metabolomics, and spatial transcriptomics data. These findings suggested that such metabolites may be associated with metabolic state changes in diverse TMEs and provided a set of candidate features for future functional investigations of tumor fatty acid metabolism.

## 4. Discussion

This study investigated metabolite abundances to reflect alterations in the energetic and material states of the tumor microenvironment. Unlike most pan-cancer studies that rely primarily on transcriptomic data to indirectly infer metabolic states [47,48,49,50,51,52,53], in this study, we focused on the metabolites themselves to provide a more direct characterization of tumor metabolic reprogramming. The widespread use of transcriptomics in cross-cohort and cross-cancer analyses is partly attributable to its technical characteristics, as sequencing platforms offer relatively stable gene coverage across studies. In contrast, metabolomics faces greater challenges in cross-study integration due to incomplete metabolite annotation and variability in experimental platforms and analytical objectives. Nevertheless, continued advances in metabolomics and spatial metabolomics have led to the accumulation of increasingly multi-layered datasets, enabling more systematic analyses at larger scales.

Leveraging these resources, we characterized tumor metabolic reprogramming across two complementary dimensions: tissue-averaged metabolic states and spatially resolved metabolic distributions (Supplementary Table S4). The differences observed between bulk and spatial metabolomic profiles highlight a crucial methodological consideration: these approaches capture complementary metabolic information at distinct organizational levels. Bulk metabolomics provides a high-sensitivity global overview suitable for quantitative tumor-normal comparisons. Still, it obscures spatial organization, whereas spatial metabolomics preserves spatial context and reveals region-specific metabolic heterogeneity masked by tissue averaging.

At the bulk level, we identified metabolic modules that exhibited both conserved alterations across multiple cancer types and cancer-specific changes. Notably, citric acid exhibited an increased abundance across all four cancer types, consistent with previous studies indicating that maintaining a certain level of intracellular citrate is essential for tumor cell survival and expansion [38]. Butyrylcarnitine has also been reported to be associated with cancer risk and progression [39,40]. Our results also found higher abundances of butyrylcarnitine in tumor tissues compared with normal tissues across multiple cancers. L-methionine was found to consistently reduce in breast, renal, and brain cancers. In agreement with our observations, previous studies have shown that elevated methionine cycle activity in tumor-initiating cells can lead to methionine consumption exceeding its regeneration [41]. In contrast, some metabolites displayed cancer-type-specific alterations, which reflect both conserved metabolic requirements and adaptations to distinct tumor-specific microenvironmental conditions.

At the spatial level, spatial metabolomics analysis found remarkable metabolic heterogeneity within the TME, even in one individual slice. Our analysis further revealed that metabolites belonging to the same metabolite family could exhibit distinct spatial distributions across tumor regions. Underscoring the importance of spatial resolution for capturing metabolic heterogeneity is obscured by tissue-averaged analyses. The spatial metabolic heterogeneity may arise from a heterogeneous intratumor or interorgan environment, such as the difference between tumor cores and edges [54]. In particular, some spatial metabolic patterns, like the spatial enrichment of lipid- and carnitine-related metabolites, were shared across tumor types, indicating the common spatial-niche-specific metabolic demands across cancers [16].

Comparing the remarkable metabolites identified based on bulk and spatial levels, only 34 metabolites were shared at both levels, indicating a relatively limited intersection. This limited overlap likely reflects the differences between bulk metabolomics and spatial metabolomics, which capture complementary but non-identical aspects of metabolic states. In addition, the observed discrepancy may also be influenced by a combination of intrinsic biological heterogeneity across cancer types and methodological variability, including differences in data origin such as distinct studies, experimental designs, and analytical pipelines. Notably, despite the limited intersection, we still observed that palmitoylcarnitine and oleoylcarnitine were consistently observed at relatively higher abundance in tumor samples across multiple metabolomics data types. Previous studies have demonstrated that increased mitochondrial palmitoylcarnitine countertransport can induce oxidative stress and promote apoptosis in cancer cells [55,56]. In addition, oleoylcarnitine has been reported to enhance self-renewal capacity and sphere formation in hepatocellular carcinoma cells through activation of the STAT3 signaling pathway, thereby directly contributing to hepatocarcinogenesis [57]. SLC22A5, a high-affinity carnitine transporter located at the plasma membrane, and CPT2, an enzyme localized to the inner mitochondrial membrane that catalyzes the reconversion of long-chain acylcarnitines entering mitochondria into acyl-CoA [58]. Spatial correspondence with the expression patterns of SLC22A5 and CPT2 exhibited relatively higher expression levels in specific tumor regions. Taken together, these results demonstrate that carnitine-related metabolites repeatedly exhibited altered abundance patterns across multiple analytical layers and cancer types. This recurrent co-occurrence across bulk, spatial, and blood-based metabolomics delineates a metabolic state feature consistently observed across diverse TMEs.

Several limitations should be acknowledged. This study was primarily based on differential and unsupervised analyses and therefore does not allow for causal inference. In addition, the limited number of spatial metabolomics tissue slices, heterogeneity in data sources and analytical platforms, and restricted metabolite coverage of current spatial technologies may affect the completeness and generalizability of some spatial patterns. Future studies incorporating larger, more standardized spatial metabolomics datasets and functional validation will be essential to further elucidate the roles of these metabolic features in tumor development and progression.

## 5. Conclusions

In this study, we integrated bulk and spatial metabolomics with blood-based metabolomics and spatial transcriptomics to examine metabolic alterations across multiple cancer types. Our analyses reveal that tumor metabolism exhibits both metabolic features shared across cancers and substantial spatial heterogeneity within the tumor microenvironment. Bulk metabolomics highlights both conserved and cancer-type-specific metabolic changes, whereas spatial metabolomics uncovers regionally distinct metabolic patterns that are not apparent at the tissue-averaged level. By integrating bulk and spatial data, we identified a limited set of metabolites showing concordant alterations across analytical layers, including several acyl-carnitines. These metabolites also displayed consistent trends in blood-based metabolomics and spatial associations with genes related to fatty acid metabolism. Collectively, these results underscore the complexity of cancer metabolism as a multi-scale and context-dependent system shaped by both inter-tumor diversity and intra-tumor spatial organization. Together, these findings underscore the value of multi-modal and spatially resolved metabolomics for characterizing pan-cancer metabolic landscapes and provide a resource for future studies investigating the functional and clinical relevance of shared tumor-associated metabolic features.

## Data Availability

This paper analyzes existing publicly available data accessible at METASPACE platform (https://metaspace2020.org/ (accessed on 9 October 2025)).

## Supplementary Materials

The following supporting information can be downloaded at https://www.mdpi.com/article/10.3390/metabo16020129/s1, Supplementary Figure S1: The enlarged part of SMM_1 in the heatmap of Figure 3A; Supplementary Figure S2: The enlarged part of SMM_2 in the heatmap of Figure 3A; Supplementary Figure S3: The enlarged part of SMM_4 in the heatmap of Figure 3A; Supplementary Figure S4: The spatial intensity distribution from PC-(18:1(9Z)/18:1(9Z)) in SMM_3 and PC-(15:0/16:0) in SMM_1 in Stomach_s8 slice. Supplementary Table S1: Summary of the tissue-based bulk and spatial metabolomics datasets collected for the pan-cancer analysis; Supplementary Table S2: Summary of the blood-based bulk metabolomics datasets collected for the pan-cancer analysis; Supplementary Table S3: Summary of annotation information for all metabolites in the HMDB; Supplementary Table S4: Summary of differentially abundant metabolites across bulk and spatial metabolomics.
