# Supplementary material for: Multi-Modal Metabolomics Deciphers Pan-Cancer Metabolic Landscapes and Spatial-Niche-Specific Alternations
Source: Metabolites. 2026 Feb 13;16(2):129. doi: 10.3390/metabo16020129 (PMC12943330; doi:10.3390/metabo16020129)
Supplement: Supplementary file 1 [file metabolites-16-00129-s001.zip › Supplementary_Figures.pdf]

Multi-Modal Metabolomics Deciphers Pan-Cancer Metabolic Landscapes and Spatial-Niche-Specific Alternations

Tingze Feng [1,2], Hai-Long Piao [1,2,3,*] and Di Chen [1,2,*]

1 State Key Laboratory of Phytochemistry and Natural Medicines, Dalian Institute of Chemical Physics, Chinese Academy of Sciences, 568 Lvshunzhong Road, Dalian 116051, China; tingzef@dicp.ac.cn

2 University of Chinese Academy of Sciences, Beijing 100049, China

3 Department of Thoracic Surgery, Cancer Hospital of Dalian University of Technology, Liaoning Cancer Hospital & Institute, Shenyang 110042, China

* Correspondence: hpiao@dicp.ac.cn (H.-L.P.); di.chen@dicp.ac.cn (D.C.); Tel.: +86-0411-39787236 (H.-L.P. & D.C.)

Supplementary Figures

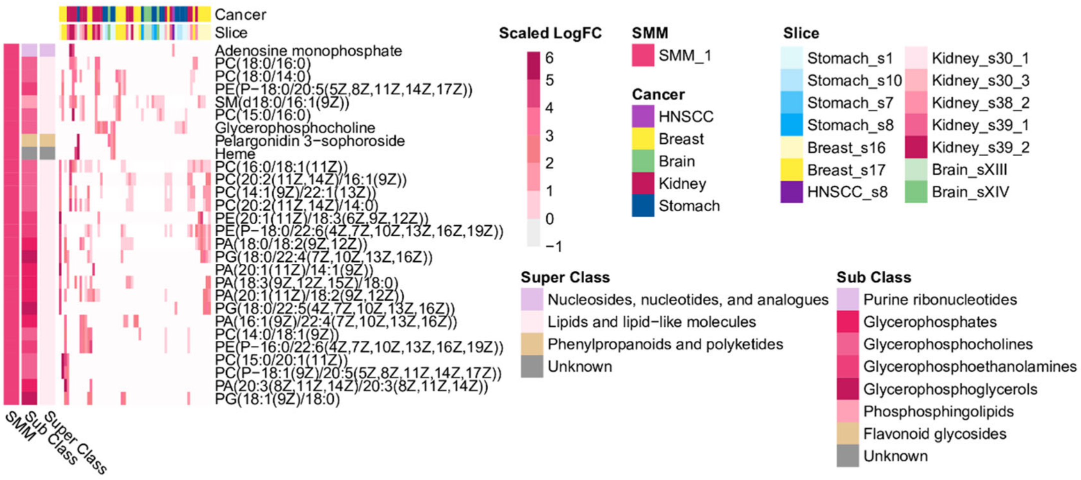

Supplementary Figure_S1. The enlarged part of SMM_1 in the heatmap of Figure 3A. Spatial clusters in this heatmap contained at least one metabolite of SMM_1 with the logFC greater than 0. HNSCC: Head and neck squamous cell carcinoma; PC: Phosphatidylcholine; PE: Phosphatidylethanolamine; SM: Sphingomyelin; PA: Phosphatidic acid; PG: phosphatidylglycerols. Glycerophospholipids are annotated based on fatty acyl composition (carbon chain length:number of double bonds), whereas sphingomyelins are annotated by sphingoid base and N-acyl chain composition.

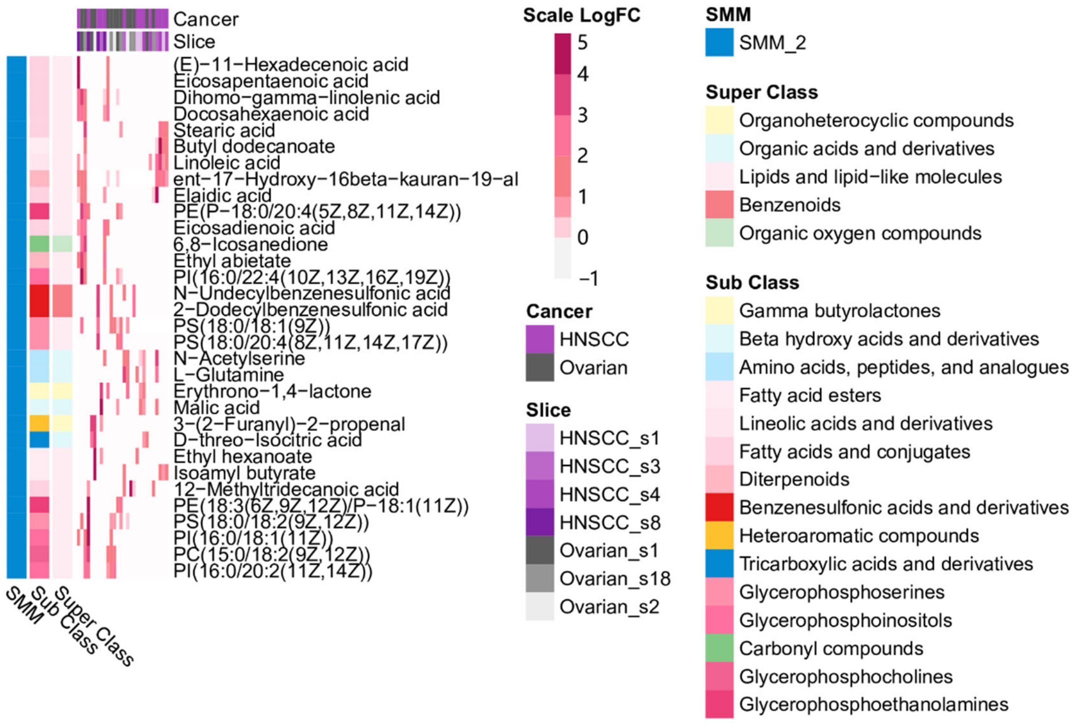

Supplementary Figure_S2. The enlarged part of SMM_2 in the heatmap of Figure 3A. Spatial clusters in this heatmap contained at least one metabolite of SMM_2 with the logFC greater than 0. HNSCC: Head and neck squamous cell carcinoma; PE: Phosphatidylethanolamine; PI: Phosphatidylinositol; PS: Phosphatidylserine; PC: Phosphatidylcholine. Glycerophospholipids are annotated based on fatty acyl composition (carbon chain length:number of double bonds).

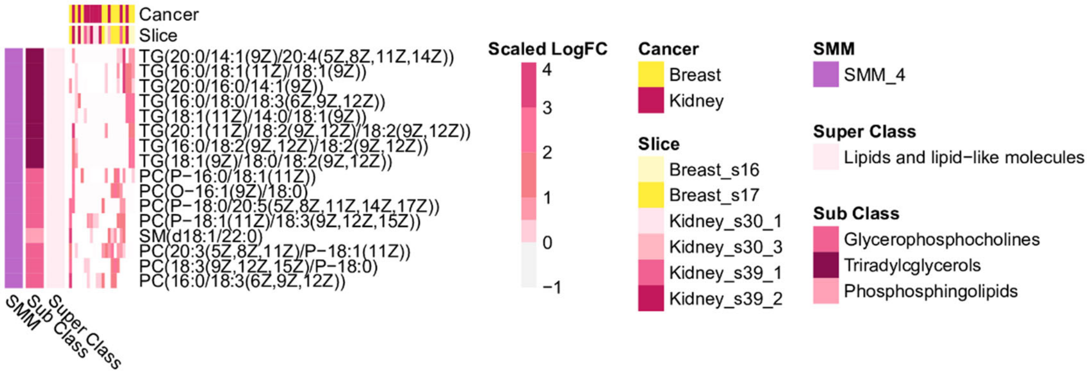

Supplementary Figure_S3. The enlarged part of SMM_4 in the heatmap of Figure 3A. Spatial clusters in this heatmap contained at least one metabolite of SMM_4 with the logFC greater than 0. TG: Triglyceride; PC: Phosphatidylcholine; SM: Sphingomyelin. Glycerolipids and glycerophospholipids are annotated based on fatty acyl composition (carbon chain length:number of double bonds), whereas sphingomyelins are annotated by sphingoid base and N-acyl chain composition.

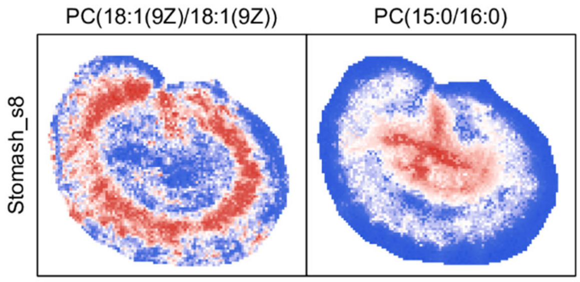

Supplementary Figure_S4. The spatial intensity distribution from PC (18:1(9Z)/18:1(9Z)) in SMM_3 and PC (15:0/16:0) in SMM_1 in Stomash_s8 slice. PC: Phosphatidylcholine. Phosphatidylcholines are annotated based on fatty acyl composition (carbon chain length:number of double bonds).
